# Beta-Glucan-Rich Extract from *Pleurotus sajor-caju* (Fr.) Singer Prevents Obesity and Oxidative Stress in C57BL/6J Mice Fed on a High-Fat Diet

**DOI:** 10.1155/2013/185259

**Published:** 2013-05-07

**Authors:** G. Kanagasabapathy, S. N. A. Malek, A. A. Mahmood, K. H. Chua, S. Vikineswary, U. R. Kuppusamy

**Affiliations:** ^1^Mushroom Research Centre, University of Malaya, 50603 Kuala Lumpur, Malaysia; ^2^Department of Biomedical Science, Faculty of Medicine, University of Malaya, 50603 Kuala Lumpur, Malaysia; ^3^Institute of Biological Sciences, Faculty of Science, University of Malaya, 50603 Kuala Lumpur, Malaysia

## Abstract

Mushrooms have been used in folk medicine for thousands of years. In this study, the effect of **β**-glucan-rich extract of *P. sajor-caju* (GE) on lipid lowering and antioxidant potential was assessed in C57BL/6J mice fed on a high-fat diet. Obesity was induced in C57BL/6J mice by feeding a high-fat diet. The control groups in this study were ND (for normal diet) and HFD (for high-fat diet). The treated groups were ND240 (for normal diet) (240 mg/kg b.w) and HFD60, HFD120, and HFD240 (for high-fat diet), where the mice were administrated with three dosages of GE (60, 120, and 240 mg GE/kg b.w). Metformin (2 mg/kg b.w) served as positive control. GE-treated groups showed significantly reduced body weight, serum lipid, and liver enzymes levels. GE also attenuated protein carbonyl and lipid hydroperoxide levels by increasing the enzymic antioxidants (SOD, CAT, and GPx) activities in the mice. GE-treated groups induced the expression of hormone sensitive lipase (HSL) and adipose triglyceride lipase (ATGL) while downregulated the expression of peroxisome proliferator-activated receptor gamma (PPAR-**γ**), sterol regulatory binding protein-1c (SREBP-1c), and lipoprotein lipase (LPL). Hence, GE prevented weight gain in the mice by inducing lipolysis and may be valuable in the formulation of adjuvant therapy for obesity.

## 1. Introduction

Obesity has reached epidemic proportions and is a major contributor to the global burden of chronic disease and disability because of its increasing prevalence in all age groups, sex, and race with the changes of lifestyles and dietary intake. A recent statistical report by the World Health Organization showed that one out of ten adults were overweight; hence, there are more than one billion overweight adults [1, 2]. Besides that, according to the National Health and Morbidity Surveys (2011), 15.1% of Malaysians aged 18 and above were obese thus Malaysia has the highest rate of obesity in south east Asia and the 6th in Asia. Obesity is a chronic metabolic disorder that results from the disequilibrium between energy intake and energy expenditure. It is characterized by enlarging fat mass and elevated lipid concentration in blood. The amount of fat mass is increased when the number and size of adipocytes are increased by proliferation and differentiation [3]. The obvious alternatives for the treatment of obesity are diet, exercise, and surgical intervention such as bariatric surgery, Roux-en-Y gastric bypass, gastric banding, and sleeve gastrectomy. However, it is proven to be successful only in a small minority of the population [4, 5]. Drugs that are currently available for the management of obesity, include orlistat (Xenical) which reduces intestinal fat absorption through inhibition of pancreatic lipase and sibutramine (Reductil), and appetite suppressant [2] which was found to cause numerous side-effects which include valvular heart disease, high blood pressure, dry mouth, constipation, and headache [6]. Multiple risk factor syndrome or metabolic syndrome such as insulin resistance [7], diabetes mellitus [8], cardiovascular disease, stroke, hypertension [9], and dyslipidemia [10] is a growing medical problem in industrialized countries. Obesity is the central and causal component in this syndrome [11]. Furukawa et al. [11] reported that in obese individuals, elevated reactive oxygen species (ROS) upregulates the expression of NADPH oxidase, establishing a vicious cycle that augments oxidative stress in adipocytes and blood circulation. The ROS will increase the expression of chemoattractants such as monochemoattractant proteins-1 (MCP-1), by-products of lipid oxidation (lipid hydroperoxides and malondialdehyde (MDA)), and protein oxidation (protein carbonyl) [12, 13] which are linked with systemic inflammation which then lead to the development of metabolic syndrome. However, it is also well reported that antioxidants can inactivate these ROS and thus prevent metabolic deregulation including metabolic syndrome [14].

Mushrooms are well known for their medicinal properties and have been widely used in traditional medicine. The medicinal effects of mushrooms include antioxidant, antiviral, antibacterial, antifungal, antiparasitic, detoxification, immunomodulatory, antitumor, radical scavengers, antiinflammatory, antihyperlipidemic, or antihypercholesterolemic, hepatoprotective, and antidiabetic [14]. In Malaysia, the genus *Pleurotus* (oyster mushroom) which has been shown to have definite nutritive (high quality proteins, vitamins, and very little lipids or starch) and medicinal values is widely cultivated. This mushroom is mostly popular in countries such as India, China, and Japan and is reported to be able to reduce the cholesterol level in blood [15] and prevent hyperglycemia, insulin resistance, and inflammation in adipose tissue [16]. *Pleurotus* mushroom is rich in fiber yet low in calories and fat, and it has been cited as a potential weight-loss aid. The dietary fibers in the mushroom consist of chitin, hemicelluloses, mannans, and *β*-glucans. Beta-glucans are polysaccharides with glucose residue linked by beta glycosidic bonds. The fermentability of *β*-glucans and their ability to form highly viscous solutions in the human gut may constitute the basis of their antiobesity benefits [17]. Natural products containing *β*-glucans have been used for thousands of years, but *β*-glucans were only identified as active components recently. Therefore, this study was undertaken to investigate the effects of *β*-glucan-rich extract (GE) from *P. sajor-caju* on prevention of obesity and oxidative stress in C57BL/6J mice fed on a high-fat diet.

## 2. Materials and Methods

### 2.1. Mushroom Samples

All necessary permits and permission for the collection of materials for the described field study were obtained, and the party involved is duly acknowledged. Fresh fruiting bodies of *Pleurotus sajor-caju* (10 kg) were grown and collected from Mr. Kuan Kek How mushroom farm in Semenyih, Selangor Darul Ehsan, Malaysia. Authentication of *P. sajor-caju* was carried out by the Mushroom Research Centre (MRC), University of Malaya, and a voucher material (KUM 50082) for this study was deposited at the MRC culture collection.

### 2.2. Isolation and Purification of GE

The isolation and purification of GE were carried out based on the method described by Roy et al. [18]. The *β*-glucan level in GE was estimated using the *β*-glucan kit (specific for mushroom and yeast) purchased from Megazyme International (Ireland). The enzyme kit contains exo-1,3-*β*-glucanase, *β*-glucosidase, amyloglucosidase and invertase, glucose determination reagent (GOPOD-glucose oxidase, peroxidase, and 4-aminoantipyrine), and glucose standard solution. The estimation of total glucan content was done by hydrolysing GE with 37% hydrochloric acid (v/v) for 45 minutes at 30°C and continued for 2 hours at 100°C. After neutralization with 2 M potassium hydroxide, glucose hydrolysis was carried out using a mixture of exo-*β*-(1-3)-D-glucanase and *β*-glucosidase in sodium acetate buffer (pH 5.0) for 1 hour at 40°C. To measure the total glucan content, glucose oxidase-peroxidase mixture was added to GE and incubated for 20 minutes at 40°C. The absorbance of the resulting colour complex was measured using a spectrophotometer (Bio-Tek Instruments Inc, USA) at 510 nm. The *α*-glucan content was estimated according to the same method as described above after enzymatic hydrolysis with amyloglucosidase and invertase. The *β*-glucan content was calculated by subtracting the *α*-glucan from the total glucan content. Glucan content was expressed as percentage (w/w) of dry weight (DW).

### 2.3. Animals and Ethics Statement

This study was conducted in conformity with the policies and procedures of the Animal Care and Use Guidelines of Faculty of Medicine, University of Malaya, with reference to the 8th edition of Guide for the Care and Use of Laboratory Animals by the Institute of Laboratory Animal Research, National Academy of Science, USA. The animal ethics approval was obtained from Animal Care and Use Committee of Faculty of Medicine, University of Malaya (IACUC, UM) (approval number: ISB/14/07/2010/GK [R]). Female C57BL/6j mice (7 weeks old) were purchased from BioLasco Laboratory, Taiwan. The animals were maintained in stainless steel wire mesh cages in a room kept at 21°C with a standard condition of 12-hour light/dark cycle (light period: 8:00–20:00 hour) with free access to food and water which were provided fresh every day.

### 2.4. Experimental Design

After one week of acclimatisation, the mice were randomly assigned (based on weight) into seven groups (*n* = 6).  Table 1 shows the type of diet and concentration of GE administered to each group. On caloric basis, the normal diet contained 5% fat, 69.2% carbohydrate, and 25.8% protein whereas the high-fat diets (TestDiet, USA) comprised 45% of fat (46.1% fat from lard, 35.8% carbohydrate, and 18.1% protein) and 60% of fat (61.6% fat from lard, 20.3% carbohydrate, and 18.1% protein). GE was administered thrice a week via epigastric route using a feeding needle (size 20) to groups ND240, HFD60, HFD120, and HFD240 for 16 weeks. In this study, metformin (2 mg/kg b.w) was used as the positive control (HFDMET) since metformin has been reported to have comparable effects with orlistat (antiobesity drug) [19], and it is also widely used to treat type 2 diabetes which is closely associated with obesity [11]. After 7 weeks of feeding with 45% of fat, the animal diet was substituted with 60% of fat for groups HFD, HFD60, HFD120, HFD240 and HFDMET. The diet for groups ND and ND240 was not altered throughout the experiment. For the normal diet group, only 240 mg/kg of body weight of GE (highest dose) was administrated to the mice in order to reduce the usage of mice.

#### 2.4.1. Sample Collection and Analytical Methods

Body weight and food consumption were monitored daily. During the experimental period, urine was collected from each group weekly (every Monday morning at 10:00 hour). At the end of the 16 weeks, the mice were anesthetized with ether after withholding food for 12 hours and were sacrificed by aortic exsanguination. Blood samples were collected in a SST glass serum tube with gold BD Hemogard closure (BD Vacutainer, USA). Serum samples were separated after centrifugation at 2400 ×g for 15 minutes. The serum samples from each mouse (within a group) were pooled together in order to have sufficient serum for further analysis. The pooled serum samples were sent to the Clinical Diagnostic Laboratory Unit, University Malaya Medical Centre, for the serum lipid and liver analysis. Immediately after blood collection, the liver and kidney were perfused *in-situ* with ice-cold saline. The weight of the liver and kidney of mice from each group were recorded. Eight mL of ice-cold phosphate buffer saline (PBS) was added to one gram of liver or kidney. The samples were then homogenized using a homogenizer (WiseMix HG-15A, Germany). Adipose tissues were removed and stored in RNAlater solution (Applied Biosystems, USA) and refrigerated at 4°C overnight. All samples were then stored at −80°C until further analysis was carried out.

### 2.5. Urinary Oxidative Indices Measurement

The protein carbonyl content (AOPP) was determined as previously described [20]. Chloramine-T solution of known concentrations (0 to 500 *μ*M) was used as a standard for the estimation of AOPP concentration, and the result was expressed as *μ*M of chloramine-T. Lipid hydroperoxide level was determined based on the method described by Esterbauer and Cheeseman [21] with modifications. 1,1,3,3-Tetraethoxypropane (TEP) solution of known concentration (2.5 to 20 *μ*M) was used as a standard for quantification, and the result was expressed as *μ*M of TEP. The DNA damage level was quantified using 8-hydroxy-2-deoxy-Guanosine (8-OHdG) EIA kit (Cayman Chemical, USA). 8-Hydroxy-2-deoxy-Guanosine hydroxyl EIA standard (10.3 pg/mL to 30 ng/mL) was used for quantification, and the result was expressed as pg/mL.

### 2.6. Enzymic Antioxidant Activity Measurement

The kidney and liver tissue homogenates were used to measure the activities of superoxide dismutase (SOD [EC-1.15.1.1]), glutathione peroxidase (GPx [EC-1.11.1.9]), and catalase (CAT [EC-1.11.1.6]). Commercially available kits were used for SOD, CAT, and GPx assays (Calbiochem, Germany). The protein content of the homogenates was determined using the Bio-Rad Protein Assay (Barcelona, Spain) [22] with bovine serum albumin as a standard. Enzyme activities were expressed in units per milligram of protein. One unit of SOD activity was defined as the amount of enzyme that exhibited 50% dismutation of the superoxide radical. One unit of CAT activity was defined as the amount of enzyme that caused the formation of 1.0 nmol formaldehyde per min. The unit of GPx activity was expressed as nanomoles of NADPH per min (calculated using an extinction coefficient of 0.00373 *μ*M^−1^).

### 2.7. Lipid Peroxidation Assay (LPO)

The LPO assay was determined according to the modified method of Kuppusamy et al. [23] based on thiobarbituric acid reaction in which MDA was used as an index of lipid peroxidation. Trichloroacetic acid (15%) and thiobarbituric acid (1%) were added to the tissue homogenates in triplicates. The mixtures were incubated in boiling water bath for 10 minutes and were centrifuged at 6000 ×g for 10 minutes to remove the sediments. The absorbance was read at 532 nm using a spectrophotometer (Bio-Tek Instrument Inc., USA). 1,1,3,3-Tetraethoxypropane (TEP) solution of known concentration (2.5 to 20 *μ*M) was used as a standard for quantification, and the result was expressed as mmol/L of TEP.

### 2.8. Gene Expression Using Real Time: RT-PCR

The total RNA was isolated from the adipose tissue using Ambion RNAqueous-Micro Kit (Applied Biosystems, USA). The purity of recovered total RNA was estimated by calculating the ratio of absorbance reading of 260 nm and 280 nm. The integrity of RNA was estimated using Agilent 2100 Bioanalyzer (Applied Biosystems, USA). Purified RNA with an *A*_260_/*A*_280_ ratio between 1.8–2.0 and RIN values 8–10 was further used to synthesize complementary DNA (cDNA) by polymerase chain reaction (PCR) approach. High Capacity cDNA Reverse Transcription Kit (Applied Biosystems, USA) which contained all reagents needed (RT buffer, dNTP mix, random primers, Multiscribe reverse transcriptase enzyme, and nuclease free water) for reverse transcription (RT) of total RNA to single-stranded cDNA was used in this study. The mixture was then loaded into a thermal cycler (Eppendorf, USA), and PCR was carried out according to optimized thermal cycling conditions provided by the manufacturer.  Table 2 shows the list of genes investigated in this study and the corresponding accession numbers. Endogenous control used in this study was eukaryotic 18S rRNA with FAM/MGB probe. All TaqMan (Applied Biosystems, USA) probes used in this investigation were labeled with FAM reporter dye at the 5′ end and a MGB quencher at the 3′ end. The quantification approach used was the comparative CT method, also known as 2^−ΔΔCt^ method [24].

### 2.9. Statistical Analysis

Data are shown as mean ± SD of triplicate assays. One-way analysis of variance was used to estimate the significant differences between groups. Statistical significance was accepted at *P* < 0.05. Duncan's multiple range tests (DMRT) was used to determine the significant differences between groups. Statgraphics Plus software (version 3.0, Statistical Graphics Corp., Princeton, NJ, USA) was used for all statistical analyses. All figures were drawn using GraphPad Prism 5 (GraphPad Software Inc., California, USA).

## 3. Results and Discussion

### 3.1. Weight and Estimation of *β*-Glucan Concentration in GE

Fresh *P. sajor-caju* (5.5 kg) was boiled for 8 hours to obtain 12.31 g of GE. The concentration of total glucan in GE was 85.95% (w/w) meanwhile the concentrations of *α*-glucan and *β*-glucan were 5.4% (w/w) and 80.55% (w/w) which corresponded to 0.01% and 1.5% in fresh mushroom, respectively [17].

### 3.2. Effects of GE on the Changes in Body Weight and Serum Lipid Levels

**Figure 1:**
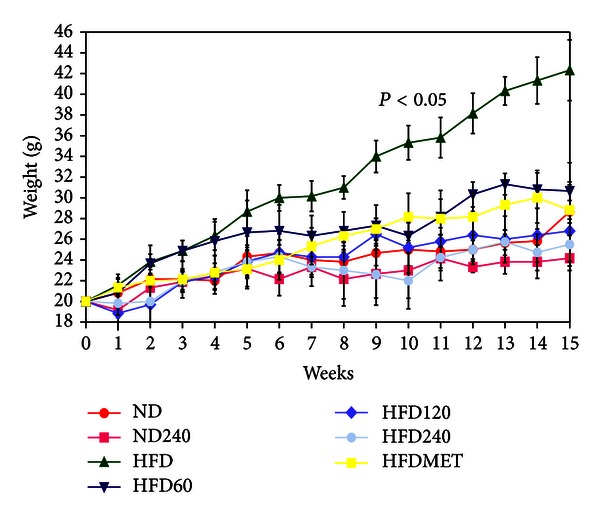
Effects of GE and metformin on body weight changes in C57BL/6J mice fed on a high-fat diet or normal diet. The concentrations of GE were 60, 120, and 240 mg/kg/day. Metformin (2 mg/kg/day) was used as positive control. Values expressed are means ± S.D of (*n* = 6 per group) measurements.

The test compounds (GE/metformin/vehicle) were only administered thrice a week to the mice in order to avoid physical stress. The mean food consumption was not significantly different between high-fat diet-treated mice and high-fat diet plus GE-treated mice.  Figure 1 shows the effects of GE and metformin on body weight changes in the mice. The body weight in the ND group gradually increased during the 16-week period. In contrast, the body weight of mice in the HFD group showed a rapid increase of body weight. The descending order of the percentages of weight gain in each group was HFD > HFD60 > HFD120 > HFDMET > ND > HFD240 > ND240. The mice in HFD60, HFD120, and HFD240 groups had 27.55%, 36.69%, and 39.76% lower body weight, respectively, compared to HFD group. HFDMET group showed 31.90% lower body weight compared to HFD group; hence, the potential weight lowering effect of GE-treated groups were comparable to HFDMET group. Obesity has been associated with increased triglycerides (TG), very low-density lipoprotein (VLDL), total cholesterol (TC), and decreased high-density lipoprotein cholesterol (HDL-c) and thus is also a risk factor of cardiovascular disease [25].  Table 3 shows the serum lipid profile which includes the levels of TG, TC, HDL-c, low-density lipoprotein cholesterol (LDL-c), and atherogenic index (AI). In HFD control group, the TG level was increased by 33.3%, TC increased by 40%, HDL-c increased by 34.6%, and LDL-c increased by 171.4% compared to those in the ND group, thus the mice in HFD were considered to be hyperlipidemic. Meanwhile, mice in HFD60, HFD120, and HFD240 groups showed considerably reduced levels of TG, TC, and LDL-c compared to the HFD group, and this effect was dose dependent. The percentages of reduction for TG, TC, and LDL-c levels in HFD60 were 12.5%, 7.1%, and 60.5%, respectively. The percentages of reduction for TG, TC, and LDL-c levels in HFD120 were 25%, 10.7%, and 81.6%, respectively. The percentages of reduction for TG, TC, and LDL-c levels in HFD240 were 25%, 25%, and 94.7%, respectively. However, there were no significant differences (*P* > 0.05) in the HDL-c level between the treated groups and control group. The HFDMET group showed decreased levels of TG (37.5%), TC (7.1%), and LDL-c (52.65%) levels and increased level of HDL-c (1.2%) compared to the HFD group. The AI and cardiac risk factor were calculated based on the measurement obtained from the lipid analysis. The AI was defined by TC minus HDL-c divided by HDL-c, whilst the cardiac risk factor was calculated as TC divided by HDL-c [26]. In this study, the AI risk predictor indices for the HFD group were increased compared to those in ND and GE or metformin-treated groups. In accordance to the high AI risk factor, the cardiac risk factor was also elevated in the HFD group compared to those in ND and GE or metformin-treated groups. The reductions in the atherogenic and cardiac risk indexes in GE-treated groups indicate a decreased risk of cardiovascular disease [27]. Beta-glucan has been shown to decrease LDL-c and increase HDL-c to alleviate possibly dyslipidemia and reduce cardiovascular disease [28]. Oats were first found to have a cholesterol-lowering effect, and the active component was identified as beta-glucans [29]. Similar serum cholesterol-lowering activity was also observed in Maitake, Shiitake, and Enokitake mushrooms [30]. The mechanism for LDL-c lowering by *β*-glucans is speculated to involve bile acid binding. The increased exclusion of bile acids activates cholesterol 7*α*-hydroxylase and upregulates low-density lipoprotein receptor (LDLR) and thus increases the transport of LDL-c into hepatocytes and the conversion of cholesterol into bile acids [31].

### 3.3. Effects of GE on Liver Enzymes

Table 4 shows the effects of GE and metformin-treated groups on liver enzymes. Increased liver enzyme concentrations and activity in the serum are conventionally interpreted as a marker of liver damage. In this study, the alanine transaminase (ALT), aspartate transaminase (AST), and alkaline phosphate (ALP) levels of mice in the HFD group were significantly elevated compared to the other groups. However, there were no changes in the glutamyl transferase (GGT) level between these groups. A recent study demonstrated that obese patients with increased serum TG level showed raised levels of each of the four liver enzymes [32]. Weight reductions have been shown to reduce the liver enzyme levels [33]. The present study shows that GE confers protection against high-fat diet-mediated liver damage.

### 3.4. Effects of GE on the Urinary Oxidative Indices

**Figure 2:**
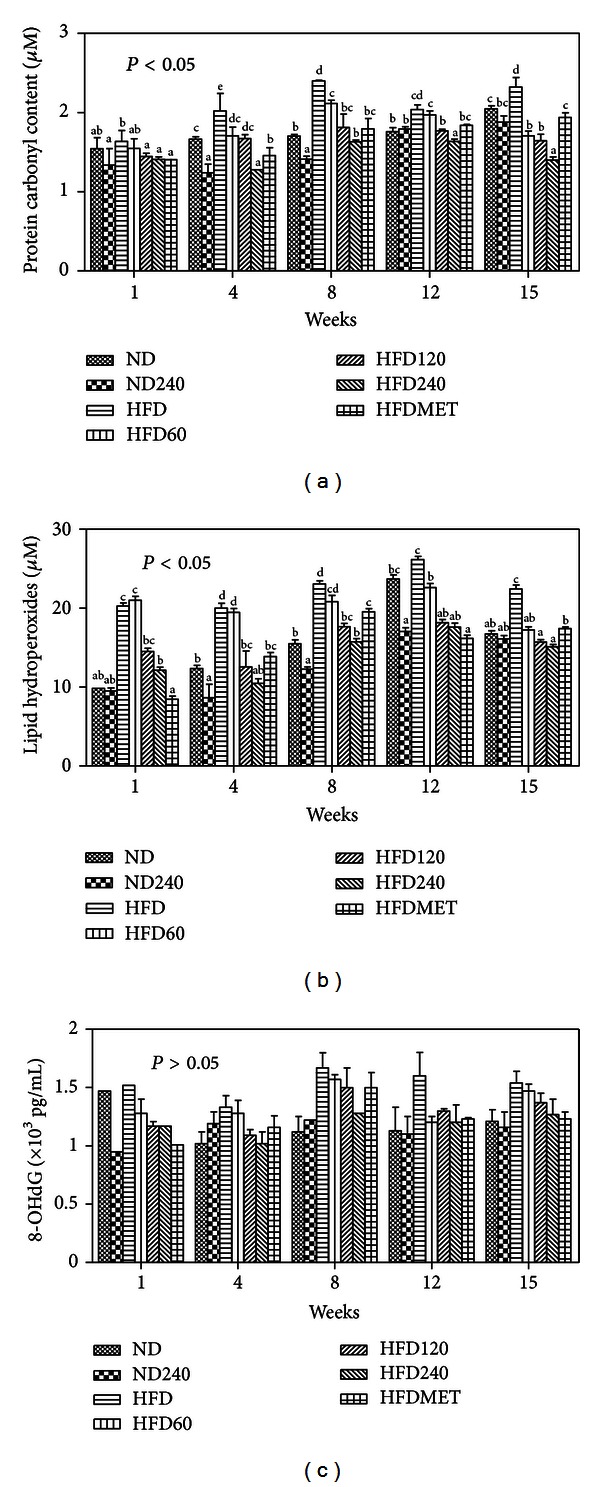
Effects of GE and metformin on (a) AOPP (b) lipid hydroperoxide, and (c) 8-OHdG levels in urine samples of C57BL/6J mice fed on a high-fat diet or normal diet. Values expressed are means ± S.D of triplicate measurements (*n* = 6 per group). For same assay with various treatment groups, superscripts in the different bar with different alphabets (a)–(e) were significantly different (*P* < 0.05). Superscripts with same alphabets were not significantly different between the treated groups (*P* > 0.05). There was no significant difference observed in the 8-OHdG levels between the groups tested (*P* > 0.05).

Oxidation products can be found in the urine and are considered to reflect local and systemic oxidative stress [34]. Figures 2(a)–2(c) show the AOPP, lipid hydroperoxide, and 8-OHdG levels in each group during the 16 weeks of experiment. The AOPP, lipid hydroperoxides, and 8-OHdG levels in the ND group gradually increased every week, however, these oxidative stress indices were significantly elevated in the HFD group compared with all other groups. The mice in HFD group were obese, and this may have contributed to the increased level of oxidative stress indices in the animals [35]. The AOPP and lipid hydroperoxide levels in GE-treated groups were lower compared to the HFD group, and this effect was dose dependent. Similarly, HFDMET also showed a decrease in AOPP and lipid hydroperoxide levels compared to the HFD group. The 8-OHdG level was elevated in HFD group, however, no significant differences were observed between all the groups tested (*P* > 0.05). Studies have shown that elevated levels of MDA [36], AOPP [37], and 8-OHdG [38] in obese animals or humans are associated with several disease conditions including hypertension, diabetes, cardiovascular diseases, and renal diseases [39].

### 3.5. Effects of GE on Enzymatic Antioxidant Levels in Liver and Kidney Homogenates

Fruits, vegetables, spices, herbs, and mushrooms have been studied for their antioxidant properties *in-vitro* extensively [40, 41]. However, the demonstration of the antioxidant properties of these components *in-vivo* is scarce but is gaining importance nowadays. Previously, antioxidant capacity has been mainly assessed in serum or plasma after an oral intake of a food infusion. Nevertheless, numerous studies have also suggested that oxidative processes occurring in various tissues and organs in the human body may be crucial in the onset of metabolic diseases [42]. It is reported that, after absorption, the antioxidant compounds are transferred through the blood circulation to various organs [43]. In the present study, the enzymic antioxidant activities and LPO level were assessed in the liver and kidney homogenates (Table 5), since these are the key organs in the mammalian oxidative metabolism. The natural antioxidant system consists of a series of antioxidant enzymes and numerous endogenous and dietary antioxidant compounds that react with and inactivate ROS. The primary antioxidant enzymes include SOD, CAT, and GPx. Meanwhile, the nonenzymatic antioxidants include vitamin C, vitamin E, *β*-carotene, reduced glutathione (GSH), and numerous phytochemicals. Cells must maintain their levels of antioxidants, often defined as their antioxidant potential, through dietary intake and/or de novo synthesis [44, 45]. Increased levels of ROS in cells and tissues may act as a signal to enhance the activity and expression of antioxidant enzymes. A high-fat diet is known to increase the superoxide anion (O_2_^•–^) radicals in the body. Superoxide dismutase converts the O_2_^•–^ radicals to hydrogen peroxide (H_2_O_2_) which in turn is converted to water by CAT and GPx. In this study, the HFD group showed reduced levels of SOD, CAT, and GPx activities in the kidney and liver homogenates compared to the ND group. Whereas, GE- and metformin-treated groups showed increased levels of SOD, GPx, and CAT activities compared to the control groups (ND and HFD) (Table 4). Overall, the increased level of antioxidant enzyme activities in GE- and metformin-treated groups conferred protection against oxidative damages in the mice, and this speculation is supported by the attenuated levels of oxidative stress indices such as AOPP and lipid hydroperoxide levels in the urine as well as MDA level in the kidney and liver homogenates.

### 3.6. Effects of GE on the Expression of Differentiation and Lipolysis Genes in Adipose Tissue

Adipose tissue is a complex and active secretory organ that both sends and receives signals that modulate energy expenditure, appetite, insulin sensitivity, endocrine function, inflammation, and immunity [46].  Table 6 shows the expression of the selected genes involved in the differentiation and lipolysis processes in adipose tissue. The mice fed on a high-fat diet (HFD group) weighed more and developed substantially more adipose tissue than the mice on a normal diet (ND group) (Figure 1). The mice became hyperlipidemic, and this is typically associated with obesity [47] (Table 3). PPAR-*γ* and SREBP-1c genes are the key adipose transcription factors that play important roles in lipogenesis [48]. These genes act cooperatively and sequentially to trigger terminal adipocyte differentiation. The PPAR-*γ* is expressed selectively in the adipose tissues, and it promotes the differentiation and proliferation of the preadipocytes thereby causing an increase in fat mass [49], while SREBP-1c controls the production of endogenous ligands for PPAR-*γ* as a mechanism for coordinating the actions of these adipogenic factors [48] and has been implicated as being a key regulator for fatty acid and triglyceride synthesis [50]. Meanwhile, LPL is the key enzyme that regulates the disposal of lipid in the body, and its role is to hydrolyse triglyceride circulating in the lipoprotein particles in order to facilitate the uptake fatty acids into the cells [51]. GE-treated groups had lower expression of PPAR-*γ*, SREBP-1c, and LPL compared to HFD group. PPAR-*γ* protein binds to the promoter regions of adipocyte-expressed LPL gene [52], and the attenuation of PPAR-*γ* gene expression in GE-treated groups could have attributed to the reduced expression of LPL as well. HSL and ATGL genes are reported to play an important role in the mobilization of stored triacylglycerol (TAG) [53]. The activation of these genes leads to mobilization of TAG to form glycerol and fatty acids where HSL mainly breaks down TAG to form diacylglycerol (DAG) whilst ATGL breaksdown DAG to form monoacylglycerol (MAG). Subsequently, MAG is converted to free fatty acids and glycerol by monoacylglycerol lipase (MGL) [54]. The GE-treated groups had significantly upregulated expressions of HSL and ATGL genes, and the effect was dose dependent. Therefore, it is feasible to suggest that the reduced weight gain in the high-fat diet fed mice treated with GE was due to the reduced adipose differentiation and increased lipolysis in adipocytes.

## 4. Conclusion

Previous studies have demonstrated that the lipid lowering potential of *β*-glucans was mainly mediated by either bile acid binding, delay in the digestion/absorption of fat, or suppressed appetite. However, in this study, GE prevented weight gain and hyperlipidemia in C57BL/6J mice fed on a high-fat diet by inducing lipolysis and inhibiting the differentiation of adipocytes. GE also prevented oxidative stress caused by obesity by increasing the enzymic antioxidant activities, hence, GE could serve as a potential candidate for the management of obesity.

## Figures and Tables

**Table 1:** Type of diet and concentration of GE/metformin administrated to each group.

Type of diet	Groups	Treatment
Normal diet	ND	Normal diet only + saline
	ND240	Normal diet + 240 mg/kg of body weight GE

High-fat diet	HFD	High-fat diet only + saline
	HFD60	High-fat diet + 60 mg/kg of body weight of GE
	HFD120	High-fat diet + 120 mg/kg of body weight of GE
	HFD240	High-fat diet + 240 mg/kg of body weight of GE
	HFDMET	High-fat diet + 2 mg/kg of body weight of metformin

**Table 2:** Genes investigated.

Number	Gene name and abbreviation	Assay ID	Accession number
1	Adipose triglycerides lipase (ATGL/Pnpla2)	Mm 00503040_m1	NM_025802
2	Hormone sensitive lipase (HSL/Lipe)	Mm 00495359_m1	NM_001039507
3	Lipoprotein lipase (LPL)	Mm 00434770_m1	NM_008509.2
4	Peroxisome proliferator-activated receptor *γ* (PPAR-*γ*)	Mm 01184322_m1	NM_011146
5	Sterol regulatory binding protein (SREBP-1c)	Mm 00550338_m1	NM_011480.3

General abbreviation of genes selected for this study and corresponding assay ID and accession number was obtained from the Applied Biosystems website and NCBI database. Assay ID refers to the Applied Biosystems Gene Expression Assays inventoried kits with proprietary primer and TaqMan probe mix. Assay ID with “Mm” is referred to as “*Mus musculus*.” All Gene Expression Assay kits indicated are FAM/MGB probed.

**Table 3:** Effects of GE and metformin on lipid profile and AI in C57BL/6J mice fed on a high-fat diet or normal diet.

Groups	Serum concentration (mmol/L)				
	TG	TC	HDL-c	LDL-c	AI
ND	0.60 ± 0.02^ab^	2.03 ± 0.5^a^	1.87 ± 0.05^a^	0.14 ± 0.0^ab^	0.07
ND240	0.70 ± 0.02^b^	1.80 ± 0.4^a^	1.79 ± 0.01^a^	0.01 ± 0.0^a^	0.01
HFD	0.8 ± 0.2^bc^	2.80 ± 0.3^b^	2.52 ± 0.3^b^	0.38 ± 0.1^c^	0.11
HFD60	0.70 ± 0.1^b^	2.60 ± 0.2^b^	2.35 ± 0.2^b^	0.15 ± 0.0^ab^	0.11
HFD120	0.60 ± 0.1^ab^	2.50 ± 0.2^ab^	2.41 ± 0.3^b^	0.07 ± 0.0^a^	0.07
HFD240	0.60 ± 0.4^ab^	2.10 ± 0.1^a^	2.35 ± 0.2^b^	0.02 ± 0.0^a^	0.02
HFDMET	0.50 ± 0.0^a^	2.60 ± 0.3^b^	2.55 ± 0.3^b^	0.18 ± 0.0^ab^	0.02

Values expressed are means ± S.D of (*n* = 6 per group) measurements. For same assay with various treatment groups, superscripts in the different bar with different alphabets (a–c) were significantly different (*P* < 0.05). Superscripts with same alphabets were not significantly different between the treated groups (*P* > 0.05). TG is triglycerides; TC is total cholesterol; HDL-c is high-density lipoprotein cholesterol; LDL-c is low-density lipoprotein cholesterol; AI is atherogenic index.

**Table 4:** Effects of GE and metformin on liver enzymes in C57BL/6J mice fed on a high-fat diet or normal diet.

Groups	Liver enzymes (mmol/L)			
	Alanine transaminase (ALT)	Aspartate transaminase (AST)	Alkaline phosphate (ALP)	G-glutamyl transferase (GGT)
ND	45 ± 1.2^c^	182 ± 11.2^b^	39 ± 1.2^a^	<3
ND240	29 ± 1.1^a^	148 ± 10.2^a^	30 ± 1.1^a^	<3
HFD	48 ± 1.4^c^	210 ± 8.2^c^	54 ± 0.9^c^	<3
HFD60	46 ± 2.3^c^	194 ± 12.1^b^	44 ± 1.4^b^	<3
HFD120	32 ± 2.4^b^	181 ± 10.1^b^	38 ± 1.3^a^	<3
HFD240	27 ± 1.4^a^	176 ± 9.7^ab^	35 ± 1.3^a^	<3
HFDMET	39 ± 1.33^b^	189 ± 9.5^b^	39 ± 1.5^a^	<3

GE concentrations were 60, 120, and 240 mg/kg/day. Metformin (MET) is used as positive control. Values expressed are means ± S.D of triplicate measurements. For same enzyme level with various treatment groups, superscripts in the different bar with different alphabets (a–c) were significantly different (*P* < 0.05). Superscripts with same alphabets were not significantly different between the treated groups (*P* > 0.05).

**Table 5:** Effects of GE on enzymic antioxidants and MDA levels in the kidney and liver homogenates of C5BL/6J mice fed on a high-fat diet.

Groups		Antioxidant activity (nmol/min/mg protein)			
		GPx	CAT	SOD (U/mg protein)	LPO (mmol/L)
ND	Kidney Liver	71.08 ± 6.3^cd^ 83.95 ± 10.8^b^	103.93 ± 4.5^d^ 29.96 ± 3.9^c^	0.34 ± 0.0^d^ 0.17 ± 0.0^b^	0.89 ± 0.01^e^ 0.76 ± 0.03^b^

ND240	Kidney Liver	82.72 ± 6.9^d^ 90.42 ± 11.7^b^	112.96 ± 4.6^e^ 32.57 ± 6.7^d^	0.37 ± 0.0^d^ 0.32 ± 0.0^d^	0.83 ± 0.04^d^ 0.7 ± 0.1^ab^

HFD	Kidney Liver	32.31 ± 3.2^a^ 45.22 ± 5.5^a^	52.64 ± 1.2^a^ 15.18 ± 8.7^a^	0.017 ± 0.0^a^ 0.01 ± 0.0^a^	0.9 ± 0.04^e^ 0.92 ± 0.1^c^

HFD60	Kidney Liver	32.46 ± 4.9^a^ 68.20 ± 8.3^b^	79.72 ± 1.3^b^ 22.98 ± 5.7^b^	0.23 ± 0.0^b^ 0.19 ± 0.0^b^	0.86 ± 0.02^de^ 0.62 ± 0.1^a^

HFD120	Kidney Liver	36.16 ± 5.1^ab^ 74.86 ± 8.7^b^	86.40 ± 11.6^bc^ 24.91 ± 9.7^bc^	0.27 ± 0.0^c^ 0.23 ± 0.0^bc^	0.64 ± 0.03^b^ 0.6 ± 0.04^a^

HFD240	Kidney Liver	44.58 ± 5.3^b^ 61.39 ± 8.9^b^	84.29 ± 1.5^bc^ 24.30 ± 1.1^bc^	0.22 ± 0.0^b^ 0.26 ± 0.0^c^	0.57 ± 0.04^a^ 0.61 ± 0.06^a^

HFDMET	Kidney Liver	66.83 ± 5.5^c^ 119.06 ± 9.3^c^	89.49 ± 2.7^c^ 25.80 ± 8.5^bc^	0.20 ± 0.0^b^ 0.19 ± 0.0^b^	0.74 ± 0.03^c^ 0.64 ± 0.05^ab^

GE concentrations were 60, 120, and 240 mg/kg/day. Metformin (MET) is used as positive control. Values expressed are means ± S.D of triplicate measurements. For same antioxidant activity with various treatment groups, superscripts in the different bar with different alphabets (a–e) were significantly different (*P* < 0.05). Superscripts with same alphabets were not significantly different between the treated groups (*P* > 0.05). GPx is glutathione peroxidase; CAT is catalase; SOD is superoxide dismutase; LPO is lipid peroxidation.

**Table 6:** Effects of GE on the expression of genes in adipose tissue.

Genes investigated	ND240	HFD60	HFD120	HFD240	HFDMET
Lipolysis					
ATGL	1.34 ± 0.34	1.78 ± 0.67^a^	6.05 ± 0.42^c^	5.69 ± 0.34^c^	3.84 ± 0.98^b^
HSL	1.98 ± 0.07	2.99 ± 0.17^a^	6.73 ± 0.42^c^	6.54 ± 0.32^c^	4.63 ± 1.16^b^

Differentiation					
LPL	−1.05 ± 0.09	−1.93 ± 0.18^a^	−1.12 ± 0.42^b^	−1.17 ± 0.47^b^	−2.22 ± 0.99^a^
PPAR-*γ*	−1.69 ± 0.19	−2.08 ± 0.69^a^	−1.69 ± 0.48^ab^	−1.02 ± 0.36^c^	−1.07 ± 0.16^c^
SREBP-1c	−1.27 ± 0.65	−1.01 ± 0.16^c^	−3.10 ± 0.44^a^	−2.25 ± 0.30^ab^	−2.30 ± 1.13^ab^

Results are expressed as fold variation over the appropriate control groups; ND240 indicates fold increase over ND (normal diet control group), and HFD60, HFD120, HFD240, and HFDMET indicate fold increase over HFD (high-fat diet control group). Fold variations less than one were expressed as negative numbers (e.g., a fold variation of 0.50 is expressed as −2.00). Values expressed are means ± S.D of triplicate measurements. Statistical significance was calculated based on the mean ΔCT values by DMRT for only mice fed with high-fat diet with or without GE. For same gene with various treatment groups, superscripts in the different bar with different alphabets (a–c) were significantly different (*P* < 0.05). Superscripts with same alphabets were not significantly different between the treated groups (*P* > 0.05).
